# Metabolic and Atherogenic Thresholds in the Severity of Erectile Dysfunction: The Dominant Role of the Triglyceride-Glucose Index

**DOI:** 10.3390/jcm15187016

**Published:** 2026-09-10

**Authors:** Omer Erdogan, Omur Memik, Mustafa Gunes, Oguz Ozden Cebeci, Murat Ustuner, Ahmed Omer Halat, Ali Kemal Uslubas, Taha Erseckin

**Affiliations:** Department of Urology, Kocaeli City Hospital, University of Health Sciences, 41000 Kocaeli, Türkiye

**Keywords:** erectile dysfunction, triglyceride–glucose index, atherogenic index of plasma, Castelli Risk Index-1, Castelli Risk Index-2, atherogenic coefficient

## Abstract

**Background:** This study aimed to investigate the association between metabolic and atherogenic indices and erectile dysfunction (ED) severity, and to compare their diagnostic performance in predicting severe ED. **Methods:** This retrospective study included 221 men with ED between January 2025 and January 2026. ED severity was assessed using the International Index of Erectile Function-5 (IIEF-5). The triglyceride–glucose (TyG) index, atherogenic index of plasma (AIP), Castelli Risk Index-1 and -2, and atherogenic coefficient (AC) were calculated from routine laboratory parameters. Correlation, logistic regression, and ROC analyses were performed. **Results:** Patients with severe ED (*n* = 57) had significantly higher triglyceride, fasting glucose, TyG, and AIP levels and lower high-density lipoprotein cholesterol (HDL) levels than those with mild-to-moderate ED (all *p* < 0.05). TyG (ρ = 0.369, *p* < 0.001) and AIP (ρ = 0.359, *p* < 0.001) were positively correlated with severe ED. In the multivariable model, the TyG index remained associated with severe ED after adjustment for AIP, age, BMI, total testosterone, and the TyG-by-testosterone interaction (OR: 4.354, 95% CI: 1.668–11.364; *p* = 0.003). Total testosterone was not independently associated with severe ED (*p* = 0.510), and the TyG-by-testosterone interaction was not significant (*p* = 0.281). ROC analysis showed that TyG had the best predictive performance (AUC: 0.743). **Conclusions:** The TyG index showed discrimination for IIEF-5-defined severe ED and may have value as a complementary metabolic risk-stratification parameter.

## 1. Introduction

Erectile dysfunction (ED) is a male sexual dysfunction defined by a persistent or recurring difficulty in attaining and/or maintaining a penile erection sufficient to permit satisfactory sexual activity [1]. From a global perspective, ED constitutes an important public health concern, with approximately 150 million men estimated to be affected worldwide, and its consequences may extend beyond the individual by adversely influencing the quality of life of both affected men and their partners [2,3]. The occurrence of ED varies considerably according to age. Among men younger than 40 years, prevalence estimates generally range from 1% to 10%; however, the frequency increases substantially in older age groups, with more than half of men aged 40–80 years reported to experience some degree of erectile impairment [2,3,4,5]. From an etiological perspective, ED can broadly be attributed to psychogenic, organic, or combined mechanisms, with organic factors estimated to account for nearly 80% of cases [4,5,6]. Within the spectrum of organic ED, vascular abnormalities represent the predominant underlying mechanism, making vasculogenic ED the most frequently encountered subtype [6,7]. The relatively small caliber of the penile arteries compared with the coronary circulation may render erectile function particularly vulnerable to early vascular alterations. Accordingly, ED has increasingly been recognized as a potential early clinical indicator of systemic vascular pathology that may become evident before overt cardiovascular disease develops [8].

The close relationship between ED and cardiovascular disease is further supported by the substantial overlap in their conventional risk profiles, particularly hypertension, diabetes mellitus, obesity, and abnormalities of lipid metabolism [7,9]. Conventional lipid measurements remain integral to routine cardiovascular risk assessment; nevertheless, indices derived from combinations of lipid parameters may provide additional information regarding atherogenic burden. In this context, the Atherogenic Index of Plasma (AIP), Castelli Risk Index-1 and -2 (CRI-1 and CRI-2), and Atherogenic Coefficient (AC) have emerged as potentially more sensitive indicators for identifying atherosclerotic changes and vascular injury than isolated conventional lipid measurements [10,11].

In addition to abnormalities in lipid metabolism, insulin resistance (IR) represents another important metabolic mechanism contributing to endothelial injury and subsequent deterioration of vascular function [12]. Among the available surrogate measures of IR, the Triglyceride-Glucose (TyG) index has attracted increasing attention because it can be readily calculated from routinely obtained laboratory parameters while remaining inexpensive and clinically accessible [13]. Previous investigations have reported relationships between ED and several of these metabolic and atherogenic biomarkers [9,12]. Nevertheless, evidence regarding their comparative and combined predictive performance in broader clinical populations remains insufficient, particularly with respect to their ability to discriminate different degrees of ED severity. Considering that atherosclerosis, endothelial dysfunction, and IR are biologically interconnected processes involved in vascular deterioration, simultaneous assessment of indices reflecting these pathways may potentially characterize the metabolic and vascular burden associated with ED more effectively than evaluation of a single biomarker alone.

Despite the reported associations of various metabolic and atherogenic parameters with ED, much of the available literature has focused on individual indices or has been based on relatively restricted patient populations. Consequently, direct comparative evidence examining several lipid-derived atherogenic indices together with a surrogate marker of IR within the same ED population remains limited. Evaluating these parameters simultaneously may therefore help clarify whether particular indices demonstrate a stronger relationship with the clinical severity of ED. Moreover, previous studies have predominantly investigated the association of these indices with the presence of ED, whereas their relationship with the clinical severity of ED has been less extensively characterized. Determining whether metabolic and atherogenic indices differ in their ability to discriminate severe ED may provide further insight into the relative contribution of insulin resistance and lipid-related abnormalities to erectile impairment. Such a comparative approach may also help identify readily available biomarkers that could complement conventional clinical assessment in men presenting with ED.

Accordingly, the present study aimed to evaluate and compare multiple metabolic and atherogenic indices, including the AIP, AC, CRI-1, CRI-2, and TyG, across different levels of ED severity. In addition, we sought to determine which of these indices provides the greatest predictive performance for severe ED and, consequently, whether any of them may serve as a practical biomarker for identifying patients with more pronounced erectile dysfunction in routine clinical practice.

## 2. Materials and Methods

### 2.1. Study Design and Population

This retrospective, single-center observational study included 221 men evaluated for erectile dysfunction (ED) at the urology outpatient clinic of Kocaeli City Hospital between January 2025 and January 2026. The study protocol was approved by the Kocaeli City Hospital Ethics Committee on June 11, 2026 (approval No. 2026-121).

Men aged ≥18 years who presented with ED, had an available International Index of Erectile Function-5 (IIEF-5) assessment, and had the clinical and laboratory data required for calculation of the investigated metabolic and atherogenic indices were eligible for inclusion. Laboratory measurements used for the calculation of these indices were obtained during the same clinical evaluation period as the ED assessment.

To minimize the potential impact of conditions that could significantly affect metabolic parameters or erectile dysfunction, patients with known diabetes or hypertension and those using antidiabetic/insulin/antihypertensive medications were excluded from the study. Additional exclusion criteria included clinical features suggestive of predominantly psychogenic erectile dysfunction, a history of pelvic surgery or pelvic radiotherapy, neurological disorders, active malignancy, active cardiovascular disease, endocrine disorders other than hypogonadism, and insufficient clinical or laboratory data necessary for study analysis.

The underlying etiology of ED was evaluated on the basis of medical history, physical examination, and biochemical assessment. To identify patients with clinical features suggestive of predominantly psychogenic ED, particular attention was paid to the preservation of nocturnal erections and erections during masturbation, as well as to the mode of symptom onset. Patients reporting preserved spontaneous or masturbation-related erections together with a sudden onset of erectile symptoms were considered to have features suggestive of a predominantly psychogenic etiology and were excluded from the study.

### 2.2. Assessment of Erectile Dysfunction

Erectile function was assessed using the validated Turkish version of the 5-item International Index of Erectile Function (IIEF-5) [13]. The questionnaire consists of five items evaluating erectile function, with a total score ranging from 5 to 25; lower scores indicate greater erectile impairment. For the purposes of the present analysis, patients with an IIEF-5 score of 5–7 were classified as having severe ED, whereas those with scores of 8–21 were categorized as having mild-to-moderate ED. Patients without ED (IIEF-5 scores of 22–25) were not included because the study population consisted exclusively of men evaluated for established ED. This classification allowed us to compare the investigated metabolic and atherogenic indices by clinical ED severity rather than by the presence or absence of ED.

### 2.3. Clinical and Laboratory Evaluation

Clinical and demographic data were retrospectively retrieved from the hospital medical records. The variables recorded for the present study included age, body mass index (BMI), smoking status, and alcohol consumption. Clinical history and available medical records were reviewed to assess relevant comorbidities and exclusion criteria.

Laboratory parameters obtained during the same clinical evaluation period as the assessment of erectile function were used for the analyses. Venous blood samples were collected in the morning between 08:00 and 10:00 a.m. after a fasting period of at least 12 h. The biochemical parameters evaluated included fasting blood glucose, triglycerides, total cholesterol, high-density lipoprotein cholesterol (HDL-C), and low-density lipoprotein cholesterol (LDL-C), which were measured using the ADVIA 1800 Clinical Chemistry System (Siemens Healthcare Diagnostics Inc., Tarrytown, NY, USA). LDL-C values were obtained directly from the laboratory analyzer and were not calculated by the investigators. Hormonal parameters, including total testosterone, follicle-stimulating hormone, luteinizing hormone, prolactin, and estradiol, were measured using the ADVIA Centaur XP Immunoassay System (Siemens Healthcare Diagnostics Inc., Tarrytown, NY, USA).

Only laboratory measurements available from the same clinical evaluation period as the ED assessment were considered for the present analysis.

The metabolic and atherogenic indices investigated in the present study were calculated from fasting glucose and lipid measurements obtained during the same clinical evaluation period as the IIEF-5 assessment. The indices were calculated using the following formulas:

- Triglyceride–Glucose (TyG) index: ln [fasting triglycerides (mg/dL) × fasting glucose (mg/dL)/2]
- Castelli Risk Index-1 (CRI-1): total cholesterol/HDL-C
- Castelli Risk Index-2 (CRI-2): LDL-C/HDL-C
- Atherogenic Coefficient (AC): (total cholesterol − HDL-C)/HDL-C
- Atherogenic Index of Plasma (AIP): log10 (triglycerides/HDL-C)

### 2.4. Statistical Analysis

Statistical analyses were performed using IBM SPSS Statistics for Windows, version 21.0 (IBM Corp., Armonk, NY, USA). The distribution of continuous variables was assessed using the Shapiro–Wilk test. As continuous variables showed deviations from a normal distribution (*p* < 0.05), they were summarized as median and interquartile range (IQR). Comparisons of continuous variables between the mild-to-moderate and severe erectile dysfunction (ED) groups were performed using the Mann–Whitney U test. Categorical variables were expressed as frequencies and percentages [*n* (%)], and between-group comparisons were performed using the chi-square test or Fisher’s exact test, as appropriate.

The associations between the investigated metabolic and atherogenic indices and ED severity were initially evaluated using univariate analyses. Candidate variables for the multivariable logistic regression model were selected based on both univariate statistical significance and a priori clinical relevance. TyG and AIP were included because they showed significant associations with severe ED in univariate analyses. Age and BMI were included a priori as clinically relevant potential confounders. Total testosterone was additionally included because of its established relationship with erectile function, and a TyG-by-testosterone interaction term was introduced to explore potential effect modification. Accordingly, the final multivariable model included TyG, AIP, age, BMI, total testosterone, and the TyG-by-testosterone interaction term. Severe ED (IIEF-5 score 5–7) was defined as the dependent outcome. Adjusted odds ratios (ORs) with corresponding 95% confidence intervals (CIs) were calculated to quantify the independent associations of the included variables with severe ED.

Receiver operating characteristic (ROC) curve analysis was performed to evaluate the ability of the investigated metabolic and atherogenic indices to discriminate patients with severe ED from those with mild-to-moderate ED. Discriminatory performance was quantified using the area under the ROC curve (AUC) with corresponding 95% confidence intervals. Pairwise comparisons between AUCs were performed using DeLong’s test for correlated ROC curves. For the TyG index, the optimal cut-off value for discriminating severe ED was determined using the Youden index. The threshold yielding the maximum Youden index was selected as the optimal cut-off, and the corresponding sensitivity and specificity were reported. All statistical tests were two-sided, and a *p*-value < 0.05 was considered statistically significant.

## 3. Results

The study population consisted of 221 men, with an overall mean age of 49 years and BMI of 27.72 kg/m^2^ (Table 1). Of these patients, 57 were classified as having severe ED, whereas the remaining 164 constituted the mild-to-moderate ED group. Univariate comparisons showed that age and BMI were comparable between the two groups, with neither parameter reaching statistical significance (*p* = 0.101 and *p* = 0.154, respectively) (Table 2).

Evaluation of the biochemical parameters according to ED severity demonstrated a distinct metabolic profile in patients with severe ED. Triglyceride concentrations, fasting blood glucose levels, the TyG index, and the AIP were significantly higher in the severe ED group than in patients with mild-to-moderate ED. Conversely, HDL cholesterol was significantly lower among patients with severe ED (all *p* < 0.05). No statistically significant between-group differences were identified for total testosterone, prolactin, total cholesterol, or LDL cholesterol levels (Table 2).

Correlation analysis using Spearman’s method further demonstrated significant positive relationships between severe ED and both the TyG index (ρ = 0.369, *p* < 0.001) and AIP (ρ = 0.359, *p* < 0.001). In contrast, neither the Castelli Risk Indices nor the AC showed a statistically significant correlation with severe ED (*p* > 0.05).

Variables were included in the multivariable logistic regression model based on both univariate statistical significance and a priori clinical relevance. In the multivariable analysis, the TyG index remained independently associated with severe ED (OR: 4.354, 95% CI: 1.668–11.364; *p* = 0.003), whereas AIP was no longer significantly associated with severe ED after adjustment (OR: 3.484, 95% CI: 0.443–27.381; *p* = 0.235). Total testosterone was not independently associated with severe ED (*p* = 0.510). To specifically examine whether testosterone modified the association between TyG and severe ED, a TyG × testosterone interaction term was included in the model; however, the interaction was not statistically significant (*p* = 0.281). These findings indicate that, within the present cohort, there was no evidence of a statistically significant modification of the TyG–ED severity association by total testosterone levels (Table 3).

**Figure 1:**
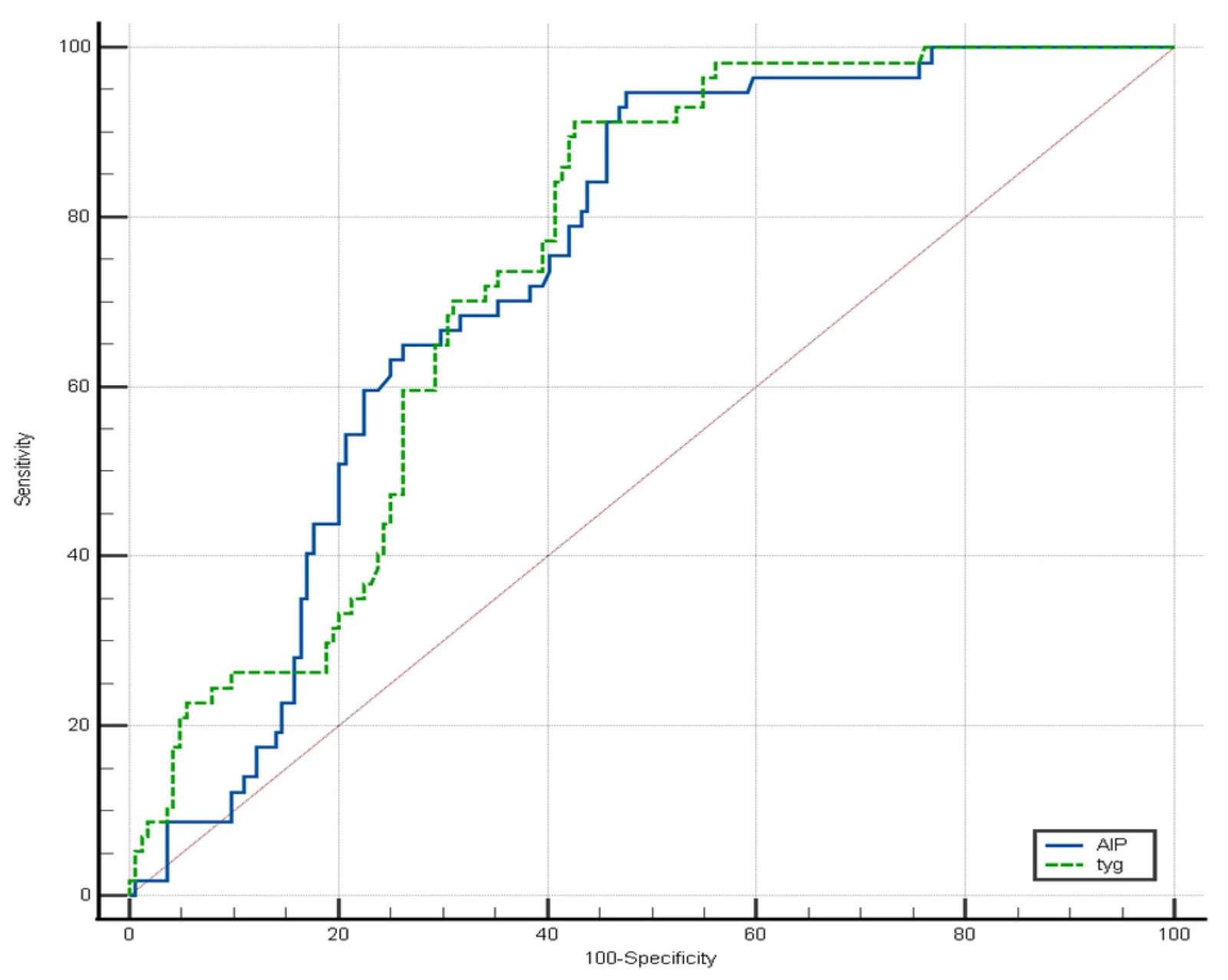
ROC curves and area under curve values of the lipid profile scores.

The discriminatory performance of the investigated lipid-derived indices was subsequently examined using receiver operating characteristic (ROC) curve analysis. Among the evaluated indices, TyG demonstrated the greatest discriminative ability, with an AUC of 0.743 (95% CI: 0.678–0.808). Pairwise comparison of the ROC curves showed that the performance of TyG was significantly better than that of the CRI-1-2 and the AC (all *p* < 0.01). By contrast, the discriminatory performances of TyG and AIP did not differ significantly (*p* = 0.842). Based on maximization of the Youden index, a TyG value of >9.16 was identified as the optimal threshold for discriminating severe ED, corresponding to a sensitivity of 91.23% and a specificity of 57.32% (Figure 1). Although the cutoff of >9.16 provided high sensitivity (91.23%), its specificity was limited (57.32%), indicating a substantial false-positive classification rate. This threshold was derived from the present cohort and was not externally validated.

## 4. Discussion

The findings of the present study demonstrate a meaningful relationship between metabolic and atherogenic profiles and the severity of ED, with the TyG index showing the strongest performance among the parameters examined. Although several lipid-derived measures, including CRI-1, CRI-2, the AC, and the AIP, have been suggested as surrogate indicators of cardiovascular risk, their ability to distinguish severe from less severe ED was generally lower than that observed for TyG in our cohort. Notably, TyG remained independently associated with severe ED after adjustment for AIP, age, BMI, and total testosterone, while also accounting for the TyG—testosterone interaction. Taken together, these observations suggest that the metabolic abnormalities represented by TyG may be more closely related to ED severity than alterations reflected solely by lipid-derived atherogenic measures.

The biological plausibility of this association is supported by the vascular mechanisms involved in erectile function. Endothelial integrity is essential for an adequate erectile response, and impairment of endothelial function constitutes a major component of vasculogenic ED. One explanation for the relationship between erectile and cardiovascular disorders is provided by the “artery size hypothesis,” according to which a comparable systemic atherosclerotic burden may become clinically evident earlier in smaller-caliber penile arteries than in larger coronary vessels [14,15]. This concept is consistent with evidence from epidemiological and clinical investigations indicating that ED may represent an early manifestation of systemic atherosclerotic disease and that ED and cardiovascular disease (CVD) share several underlying pathophysiological mechanisms [16,17,18].

Against this vascular background, considerable attention has been directed toward lipid-derived indices as potential markers of atherogenic burden. Ratios incorporating both atherogenic and anti-atherogenic lipid components, including CRI-1 and AIP, have been reported to provide cardiovascular risk information beyond that obtained from individual lipid measurements [19,20]. In relation to erectile function, Culha et al. reported significantly higher CRI-1 and AIP values among patients with severe ED and identified inverse relationships between these indices and IIEF scores, supporting a potential link between an unfavorable lipid profile and greater erectile impairment [21]. Our results partly support this relationship, particularly for AIP, which was significantly associated with severe ED in the initial analyses. However, its significance was not maintained after multivariable adjustment, whereas TyG remained independently associated with severe ED. These findings indicate that abnormalities in lipid balance may constitute only one component of the broader metabolic disturbances accompanying erectile dysfunction.

The stronger association observed for TyG may be explained by the metabolic information incorporated into this index. Unlike indices calculated exclusively from lipid parameters, TyG combines fasting triglyceride and glucose concentrations and is widely used as a surrogate measure of IR [8,22]. IR can adversely affect vascular homeostasis through several interconnected mechanisms. Reduced nitric oxide availability, enhanced oxidative stress, and persistent low-grade inflammatory activity have all been implicated in the endothelial consequences of IR [23]. Because normal penile erection depends partly on intact endothelial signaling and adequate vasodilation, these mechanisms provide biological plausibility for the observed association. However, the present study did not directly assess penile hemodynamics or endothelial function. Therefore, the TyG index should be interpreted as being associated with IIEF-5-defined ED severity rather than as a direct marker of impaired erectile vascular function.

The interplay between metabolic and hormonal factors should also be considered when interpreting the association between TyG and ED severity. Testosterone plays an important role in male sexual function, and metabolic disturbances such as insulin resistance and obesity may adversely affect the hypothalamic–pituitary–gonadal axis and testosterone homeostasis [24]. In the present cohort, total testosterone levels were numerically lower in patients with severe ED than in those with mild-to-moderate ED, although this difference did not reach statistical significance. Furthermore, total testosterone was not independently associated with severe ED in the multivariable model. To explore whether testosterone modified the relationship between metabolic dysfunction and ED severity, we additionally examined a TyG × testosterone interaction term. The interaction was not statistically significant, while TyG remained independently associated with severe ED after adjustment for total testosterone and other covariates. These findings suggest that the association between TyG and ED severity observed in our cohort was not statistically explained or modified by total testosterone levels. They support a relatively stronger statistical association of the metabolic disturbance reflected by TyG with ED severity within this specific study population.

The relationship identified in our analysis is also consistent with the growing body of evidence linking TyG to vascular abnormalities and adverse cardiovascular outcomes. Higher TyG values have previously been associated with subclinical atherosclerotic changes, increased arterial stiffness, and cardiovascular events, with some studies indicating greater predictive utility than conventional lipid measurements [25,26,27]. Evidence specifically addressing ED is also emerging. In a previous study involving 142 individuals with and without ED, TyG was identified as an independent predictor of ED [28]. Considering the substantial pathophysiological overlap among ED, IR, metabolic syndrome, and type 2 diabetes, these observations provide additional support for the potential relevance of TyG in the assessment of men with ED.

A key aspect of this research is its focus not only on the presence or absence of ED, but also on the relationship between these indices and the severity of ED. While the TyG index showed the highest AUC value among the evaluated indices, its overall discriminatory performance was modest (AUC: 0.743). A threshold value greater than 9.16 showed high sensitivity (91.23%) but limited specificity (57.32%), suggesting that a significant proportion of patients without severe ED would be classified above this threshold. Therefore, we believe that TyG, when interpreted in conjunction with clinical assessment, can serve as a complementary metabolic risk classification parameter. Previous research, including the study by Culha et al., has largely examined associations with ED or differences between predefined patient groups, whereas evidence directly comparing the ability of various metabolic and atherogenic indices to discriminate the severity of ED is limited [21]. This study, by simultaneously evaluating these parameters in the same patient population, allowed for the assessment of their relative discriminatory performance under comparable clinical conditions.

The comparatively limited performance of CRI-2 and AC in our cohort is also noteworthy. Both indices have been investigated as indicators of atherogenic and cardiovascular risk; however, neither demonstrated a significant relationship with ED severity in our analyses. Their established utility in cardiovascular risk assessment may therefore not necessarily translate into equivalent discriminatory ability across different degrees of ED. Rather than excluding a potential role for these indices in ED, our findings suggest that biomarkers developed or validated in cardiovascular populations should be specifically evaluated in men with ED before their clinical relevance is assumed.

From a clinical perspective, the current findings have practical implications because the TyG index can be calculated from fasting triglyceride and glucose measurements, which are both inexpensive and routinely available in clinical practice. Unlike specialized biomarkers or vascular investigations, its calculation does not require additional laboratory tests and therefore can provide readily accessible information about the metabolic burden associated with ED. In men presenting with ED, a high TyG value may highlight a negative metabolic profile and complement conventional clinical assessment, particularly in patients with more severe erectile dysfunction. Our findings support its potential role as an additional metabolic marker that could help identify patients for whom a more comprehensive assessment of cardiometabolic risk might be considered. Prospective studies are needed to determine whether the inclusion of TyG in routine ED assessment provides increased clinical value beyond established demographic, metabolic, and cardiovascular risk factors.

This study has several limitations. First, its retrospective and single-center design limits causal inference and may introduce selection bias. Second, ED severity was determined exclusively using the IIEF-5, a validated but subjective patient-reported instrument. Although clinical history was used to exclude patients with predominantly psychogenic presentations, the IIEF-5 does not directly assess penile hemodynamics and cannot reliably distinguish vasculogenic ED from other organic or mixed etiologies. Consequently, the observed associations with IIEF-5-defined ED severity cannot be considered direct evidence of penile arterial insufficiency, veno-occlusive dysfunction, or impaired erectile vascular function. Future prospective studies combining validated questionnaires with objective vascular assessments are needed to determine whether the TyG index and the investigated atherogenic indices are associated with objectively confirmed penile vascular impairment. Furthermore, remaining confounding factors, including lifestyle and subclinical cardiovascular disease, cannot be completely ruled out. Although patients with diagnosed diabetes mellitus and hypertension were excluded, metabolic syndrome was not systematically classified because all diagnostic components, particularly waist circumference, were not consistently available in the retrospective records. Therefore, residual confounding by undiagnosed or subclinical metabolic abnormalities cannot be excluded.

Future studies should validate these findings in larger, prospective, and multicenter cohorts that include more heterogeneous ED populations and common cardiometabolic comorbidities. External validation of the TyG threshold identified in the present study is particularly important to determine its reproducibility and clinical utility. Incorporating objective assessments of penile vascular function, such as penile Doppler ultrasonography, may also help clarify whether higher TyG values are specifically associated with vasculogenic impairment. Furthermore, longitudinal studies are needed to determine whether changes in TyG over time parallel changes in erectile function and whether improvement in metabolic status is associated with improvement in ED severity. Finally, future predictive models could evaluate whether adding TyG to established demographic and cardiometabolic risk factors provides incremental discriminatory or prognostic value beyond conventional clinical assessment alone.

## 5. Conclusions

In conclusion, among the metabolic and atherogenic indices evaluated, the TyG index showed the strongest association with severe erectile dysfunction (ED) as defined by IIEF-5; however, its discriminative performance was modest, and the specificity of the determined threshold was limited. We believe that the TyG index may have a potential role in determining the severity of ED. When interpreted in conjunction with established clinical assessment, it may play a complementary role in characterizing metabolic risk in men with ED.

## Figures and Tables

**Table 1:** Patient demographics.

Variable	Median (IQR) (*n* = 221)	
Age (years)	49 (18)	
BMI (kg/m^2^)	27.72 (6.68)	
Smoking status	No	114
	Yes	107
Alcohol consumption	No	192
	Yes	29
Testosterone (ng/dL)	402 (226)	
Estradiol (pg/mL)	29.2 (12.6)	
Prolactin (ng/mL)	9.5 (5.33)	
Total Cholesterol (mg/dL)	188 (61.5)	
HDL Cholesterol (mg/dL)	41 (14)	
Triglycerides (mg/dL)	194 (136)	
LDL (mg/dL)	112 (44)	
Fasting Blood Glucose (mg/dL; median (IQR))	98 (22)	
Castelli Risk Index 1	4.49 (1.61)	
Castelli Risk Index 2	2.66 (1.38)	
Atherogenic Coefficient	3.49 (1.61)	
Triglyceride-glucose Index (TyG)	9.23 (0.76)	
Atherogenic index of Plasma (AIP)	0.67 (0.34)	

IQR: Interquartile Range; BMI: Body Mass Index; HDL: High-Density Lipoprotein; LDL: Low-Density Lipoprotein. All variables showed non-normal distribution (Shapiro–Wilk test, *p* < 0.05); data are presented as Median (IQR).

**Table 2:** Comparison of Clinical and Biochemical Parameters in Mild–Moderate and Severe ED Groups.

Variable		Mild–Moderate ED (*n* = 164)	Severe ED (*n* = 57)	*p* Value
Age (years; median (IQR))		48.5 (17)	51 (16)	0.101
BMI (kg/m^2^; median (IQR))		27.8 (6.01)	29.39 (5.67)	0.154
Smoking status	No (*n*; %)	83 (37.6)	31 (14.0)	0.622
	Yes (*n*; %)	81 (36.7)	26 (11.8)	
Alcohol consumption	No (*n*; %)	139 (62.9)	53 (24.0)	0.111
	Yes (*n*; %)	25 (11.3)	4 (1.8)	
Testosterone (ng/dL; median (IQR)		416.00 (224.0)	362.00 (229.0)	0.128
Prolactin (ng/mL; median (IQR)		9.45 (5.48)	9.90 (5.25)	0.885
Total Cholesterol (mg/dL; median (IQR))		188.00 (62.5)	182.00 (61.0)	0.681
HDL Cholesterol (mg/dL; median (IQR))		42.00 (13.0)	39.00 (11.0)	**0.031**
Triglycerides (mg/dL; median (IQR))		175.00 (131.00)	240.00 (75.00)	**<0.001**
LDL-C (mg/dL)		110.00 (44)	114.00 (46.00)	0.736
Fasting Blood Glucose (mg/dL; median (IQR))		97.00 (17.0)	102.00 (29.0)	**0.002**
IIEF-5 Score		14.00 (6.0)	5.00 (1.0)	**<0.001**
Castelli Risk Index 1 (median (IQR))		4.35 (1.74)	4.77 (1.30)	0.116
Castelli Risk Index 2 (median (IQR))		2.57 (1.39)	2.69 (1.28)	0.105
Atherogenic Coefficient		3.35 (1.73)	3.77 (1.30)	0.116
Triglyceride-glucose Index (TyG)		9.05 (0.84)	9.45 (0.54)	**<0.001**
Atherogenic index of Plasma (AIP)		0.595 (0.36)	0.789 (0.17)	**<0.001**

All comparisons were performed using the Mann–Whitney U test (non-parametric). Bold red values: statistically significant (*p* < 0.05). Abbreviations: BMI: Body Mass Index; HDL: High-Density Lipoprotein; LDL: Low-Density Lipoprotein.

**Table 3:** Multivariable logistic regression analysis of factors associated with severe ED.

Variable	β	S.E.	OR (95% CI)	*p*
Triglyceride-glucose Index (TyG)	1.471	0.489	4.354 (1.668–11.364)	**0.003**
Atherogenic index of Plasma (AIP)	1.248	1.052	3.484 (0.443–27.381)	0.235
Age	0.017	0.017	1.017 (0.983–1.052)	0.335
BMI	−0.003	0.043	0.997 (0.917–1.084)	0.942
Total Testosterone	0.001	0.001	1.001 (0.999–1.003)	0.510
TyG × Testosterone interaction	0.003	0.003	1.003 (0.998–1.008)	0.281

OR, odds ratio; CI, confidence interval; TyG, triglyceride–glucose index; AIP, atherogenic index of plasma; BMI, body mass index. Bold values indicate statistical significance (*p* < 0.05).

## Data Availability

The original contributions presented in this study are included in the article. Further inquiries can be directed to the corresponding author.

## Abbreviations

The following abbreviations are used in this manuscript:

AC	Atherogenic Coefficient
AIP	Atherogenic Index of Plasma
BMI	Body Mass Index
CRI-1	Castelli Risk Index-1
CRI-2	Castelli Risk Index-2
ED	Erectile Dysfunction
HDL-C	High-Density Lipoprotein Cholesterol
IIEF-5	International Index of Erectile Function-5
IR	Insulin Resistance
LDL-C	Low-Density Lipoprotein Cholesterol
ROC	Receiver Operating Characteristic
TyG	Triglyceride–Glucose index
